# Early Postnatal Ethanol Exposure Has Long-Term Behavioral Consequences in Female Mice

**DOI:** 10.3390/cells15070608

**Published:** 2026-03-30

**Authors:** Elizabeth C. Plunk, MaKenna Y. Cealie, James C. Douglas, Paul D. Drew, Ania K. Majewska

**Affiliations:** 1 Department of Environmental Medicine, University of Rochester Medical School, Rochester, NY 14642, USA; elizabeth_plunk@urmc.rochester.edu; 2 Environmental Health Science Center, University of Rochester Medical School, Rochester, NY 14642, USA; 3 Department of Neuroscience, University of Rochester Medical School, Rochester, NY 14642, USA; makenna_cealie@urmc.rochester.edu; 4 Department of Neuroscience, University of Arkansas for Medical Sciences, Little Rock, AR 72205, USA; jdouglas@uams.edu (J.C.D.); drewpauld@uams.edu (P.D.D.); 5 Center for Visual Science, University of Rochester, Rochester, NY 14642, USA

**Keywords:** fetal alcohol syndrome disorders, cerebellum, myelination

## Abstract

**Background/Objectives**: Fetal alcohol spectrum disorders (FASDs) occur in nearly 5% of children in the United States and have been associated with alterations in neurological functions, neuroanatomical changes, and behavioral deficits encompassing an individual’s lifetime. Alterations in myelination have been reported in both rodent models and humans. The cerebellum is a heavily myelinated brain region, and oligodendrocyte and myelination transcripts have been reported to be altered in the cerebellum following early-life ethanol (EtOH) exposure in a mouse model. In this study, we investigated cerebellar-recruited behaviors in adult female mice that were exposed to EtOH from postnatal day (P) 4 to P9. We investigated whether changes in oligodendrocyte lineage markers were present in adulthood. **Methods**: C57BL/6J offspring received a total of 5.0 g/kg/day of either ethanol (EtOH) or saline in two separate doses delivered subcutaneously two hours apart from P4 to P9. On P21, offspring were weaned and housed with same-sex littermates throughout the duration of the study. From P60 to P90, females underwent behavioral testing including an open field test (OFT), rotarod, and balance beam. Behavior naïve littermates were euthanized on P105, and cerebella were collected for qPCR to assess oligodendrocyte lineage transcripts. **Results**: We reported that, following EtOH exposure from P4 to P9, adult female mice had increased ambulatory behaviors in the OFT and subtle changes in behavior in the rotarod and balance beam compared to saline-exposed controls. Despite the behavioral changes observed in adulthood, we found that alterations in oligodendrocyte lineage transcripts present on P10 did not persist into adulthood. **Conclusions**: Subcutaneous injection of EtOH from P4 to P9 resulted in long-term consequences in locomotor and cerebellar-recruited behaviors in female mice.

## 1. Introduction

Fetal alcohol spectrum disorders (FASDs) encompass lifelong neurological and behavioral deficits and occur in nearly 5% of children in the United States [1,2]. FASDs have been shown to be associated with neuroanatomical changes [3], which include a reduction in white matter in humans [4,5,6] and myelination defects in FASD animal models [7,8]. The cerebellum is a heavily myelinated, evolutionarily conserved brain region that is home to 50% of the neurons of the mature brain, and has a protracted development spanning late gestation continuing into the first two years of postnatal life [9,10]. The developing cerebellum appears to be vulnerable to exposure to ethanol (EtOH), as FASDs have been associated with cerebellar impairments including reduced cerebellar volume [11,12] and surface area [13], along with motor deficits and delays [14,15].

The brain growth spurt period, which occurs in the third trimester for humans [16], could be a time during which the developing brain is particularly vulnerable to EtOH. Because this brain growth spurt occurs around postnatal days (P) 4 to P9 in rodents [16,17,18], many studies have examined how EtOH exposure during this time period affects the rodent brain. The cerebellum’s protracted development and its high levels of myelination may make it especially vulnerable, because myelination occurs during the brain growth spurt [19] and is therefore sensitive to EtOH [7,8,20,21]. This suggests that cerebellar oligodendrocyte cell populations may be particularly affected by ethanol exposure during the brain growth spurt. In fact, we have previously shown that 24 h after the end of P4 to P9 exposure, there had been decreased cerebellar oligodendrocyte lineage gene expression, including oligodendrocyte progenitor cells and mature myelinating oligodendrocytes, when compared to saline-exposed littermates [22,23]. Whether these changes in gene expression persist into adulthood and correlate with changes in behaviors that involve the cerebellum is unclear; however, oligodendrocytes are not the only cell types affected by early postnatal EtOH exposure. Purkinje cell (PC) [24,25,26] and granule cell [27] loss has also been reported, resulting in lower neuronal densities in adulthood, suggesting that multiple cerebellar cell types are vulnerable to EtOH and that EtOH-induced cerebellar deficits can be persistent.

As neurodevelopmental disorders are often male-biased, there is a larger body of research focusing on the understanding neurodevelopmental insults on the male brain despite the consensus that females can present with a different phenotype [28,29]. Previously, our lab has found subtle effects of P4–P9 EtOH exposure on PC number and PC interactions with microglia that were specific to the female cerebellum [24,30]. As a result, we wanted to investigate the long-term effects of binge-level EtOH treatment on cerebellar-recruited behaviors and oligodendrocyte and myelination transcript expression levels in female mice. While our previous results showed the downregulation of oligodendrocyte- and myelination-related transcripts 24 h after the end of EtOH treatment [22], here we report that these changes could be resolved by young adulthood, resulting in a normalization of these transcripts. However, despite the resolution of gene expression changes, EtOH-exposed female mice showed long-term behavioral effects in the open field and rotarod tests, whereas the balance beam test yielded variable results.

## 2. Methods

### 2.1. Animals

C57BL/6J (Jackson Labs strain 000664; Bar Harbor, ME, USA; RRID:IMSR_JAX:000664) (9 females and 9 males) were bred under a 12 h light/dark cycle at 22 ± 2 °C with chow and water provided *ad libitum*. Males were separated from the females once pregnancy was confirmed by female weight gain. All experimental protocols were carried out in strict accordance with the University of Rochester Committee on Animal Resources and National Institutes of Health guidelines.

### 2.2. Ethanol Dosing

**Figure 1:**
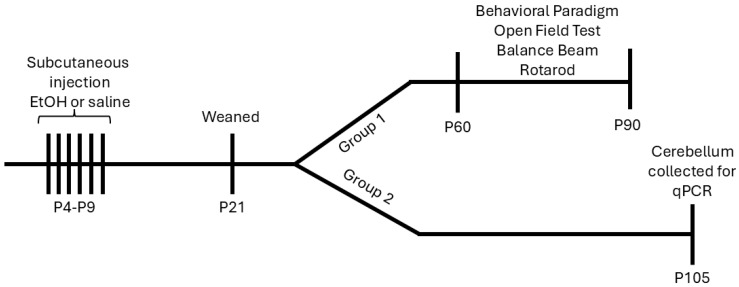
Experimental timeline. Pups received subcutaneous injections of EtOH or saline twice a day from P4 to P9. On P21, animals were weaned and housed with same-sex littermates and aged to young adulthood (P60) where they underwent a behavioral paradigm, including an open field test, balance beam, and rotarod over a period of three weeks. On P105, animals were euthanized and the cerebellum was collected for qPCR.

Postnatal day (P) 0 was denoted as the day that pups were found in the cage. From P4 to P9, all pups from nine litters were subcutaneously dosed with either ethanol (EtOH) solution or saline, allowing for within-litter controls. EtOH was provided as a total of 5.0 g/kg/day, with two separate doses of 2.5 g/kg given two hours apart (Figure 1). This exposure has been validated in previous studies producing blood ethanol concentrations (BECs) of ~450 mg/dL after the second injection on P4 and P9 [30,31]. While this is well above the lower limit of 80 mg/dL BAC, which has been used to define binge exposure in mice [32], it is generally accepted that higher doses are needed in mice to achieve the effects elicited by alcohol in humans due to allometric considerations, as well as the mouse’s faster metabolism of EtOH. In fact, a recent study calculated that doses of 3–6 g/kg in mice yielded BECs and corticosterone profiles that were not unusual in human binge drinkers [33]. At the completion of the exposure paradigm, offspring remained with the dam until P21 when they were weaned and housed with same-sex littermates that also received EtOH or saline administration. Beginning at P60, a subset of females underwent behavioral assays which were conducted between 9 a.m. and 12 p.m. by the same investigator, and analysis was performed blind to the treatment. The order of the behavior assays performed were as follows: open field test, balance beam, and rotarod. Behavior test naïve female littermates were euthanized and whole cerebella were collected for qPCR at P105.

### 2.3. Behavior

#### 2.3.1. Open Field Test (OFT)

Locomotor activity was measured across 60 minute (min) test sessions in automated chambers equipped with 48-channel infrared photobeams (Med Associates, St. Albans, VT, USA). Photobeam breaks were recorded in 5 min bins to assess horizontal, vertical, and ambulatory movements across time (resulting in 12 bins for a 1 hour locomotor activity session). Ambulatory counts were defined as the number of beam breaks in ambulatory movement. The total ambulatory time referred to the time in ambulatory movement, characterized by breaks in the *x*- and *y*-axes, and ambulatory distance represents the Euclidean distance of all ambulatory episodes. Resting time was defined as the time spent with no new photobeam breaks in the *x*-, *y*- or *z*-axes. Stereotypic time was defined as the time spent performing repetitive behaviors, like grooming or sniffing.

#### 2.3.2. Balance Beam

One day before testing began, mice were introduced to the balance beam apparatus. Each mouse was placed on the starting platform (18 cm height) and encouraged to walk across the 1 m beam (5 cm width) to a dark box. The animal was given at least 30 s to habituate to the dark box before being returned to its home cage. A smooth fabric attachment was placed approximately halfway between the beam and the countertop to prevent injury from slipping off the beam. Testing consisted of one trial per day, for three consecutive days, for each of the four beam thicknesses (5 cm, 2.5 cm, 1.25 cm, 0.63 cm). The beams were always tested in the same order from thickest to thinnest. A camera was set up perpendicular to the beam, and a video was taken each time animals crossed the beam. Foot slips were determined manually.

#### 2.3.3. Rotarod

For rotarod performance, an acceleration protocol was utilized with a starting speed of 4 mph, which was then increased by 4 mph at 15 s intervals to a final value of 40 rpm. The time for the mouse to fall from the rotarod and the speed at which this occurred were recorded. A total of three trials were carried out for each mouse in each session, with a 120 s inter-trial interval between each trial. Sessions were conducted for 5 consecutive days.

### 2.4. Isolation of RNA and cDNA Synthesis

Animals were euthanized with an overdose of sodium pentobarbital and perfused transcardially with PBS containing 5 U/mL of heparin. Following perfusion, the brain was removed, the cerebellum was separated from the forebrain and brain stem, which was then cut in half, flash frozen, and stored at −80 °C. The preparation and execution of isolation of RNA and cDNA synthesis and PCR have been previously described [24,34]. Briefly, the frozen cerebellum was homogenized using a PowerLyzer 24 homogenizer (Qiagen, Germantown, MD, USA; #13155) in the presence of 0.5 mm glass beads (Qiagen, Germantown, MD, USA; #13116-50) for 30 s at 3500 rpm, cooled on ice, and then repeated for an additional 30 s. RNA was isolated using an RNeasy Mini Kit and DNA was removed with DNAseI on-column DNA digestion according to the manufacturer’s instructions (Qiagen, Germantown, MD, USA; # 74104 and #79254). The concentration of total RNA was quantified using a NanoDrop 2000 spectrophotometer (ThermoFisher Scientific, Wilmington, DE, USA; RRID:SCR_018042) and cDNA was prepared from the mRNA template using the iScript^TM^ system (Bio-Rad, Hercules, CA, USA; # 1708891). An RNA template of 2 μg was diluted to 100 ng/μL in 20 μL of nuclease-free water and combined with 20 μL of iScript master mix (8 μL iScript 5X reaction mix, 2 μL iScript reverse transcriptase, and 10 μL nuclease-free water) for a final RNA concentration of 50 ng/μL. The reaction mixture was placed into a thermocycler and run at 25 °C for 5 min, 46 °C for 20 min, 95 °C for 1 min, and then held at 4 °C. Upon synthesis completion, the cDNA was diluted to 25 ng/μL with nuclease-free water and stored at 4 °C.

### 2.5. Quantitative Real-Time PCR Analysis

mRNA levels were quantified by real-time PCR (rtPCR) using a Bio-Rad CFX Opus 96 Real-time PCR Detection system and TaqMan^®^ Gene Expression Assays (ThermoFisher Scientific, Wilmington, DE, USA; #4331182). rtPCR was performed in duplicate 20 μL reactions using an input of 25 ng single-stranded cDNA per reaction for each sample. Each reaction contained 10 μL of 2X SsoAdvanced Universal Probes Supermix (Bio-Rad Laboratories, Hercules, CA, USA; #1725285), 1 μL of 20X TaqMan FAM-MBG Primer/Probes, 25 ng/μL of cDNA template, and 8 μL of nuclease-free water. Reactions were run in 96-well PCR plates (Bio-Rad Laboratories, Hercules, CA, USA; #HSP9601B) for 40 cycles at 95 °C for 5 s and 60 °C for 10 s following a hot start of 95 °C for 30 s. Baseline threshold CT values were obtained for each reaction and expressed as the mean ∆CT relative to the mean duplicate β-actin control reactions for each sample.

### 2.6. Statistical Analysis

GraphPad Prism software (San Diego, CA, USA; RRID:SCR_002798) was used for all statistical analysis and data visualization. All analyses were performed with the experimenter blinded to conditions. In total, 10 saline-treated and 11 EtOH-treated females were subjected to all behavioral testing, and two-way ANOVAs were performed. In OFT and rotarod analyses, all animals were represented. In the balance beam, outliers detected by ROUT outlier analysis were excluded from final analyses. *T*-tests were performed for all qPCR comparisons (N = 8–9). All sample sizes and exclusions are shown in Table 1. Detailed statistics are provided in figure legends with graphs showing the mean ± SEM, and statistical significance is defined as α < 0.05.

## 3. Results

We investigated whether high EtOH exposure from postnatal day (P) 4 to P9, the human third trimester equivalent in mice, has long-term consequences on oligodendrocyte lineage (OL) cell-related gene expression in the cerebellum and cerebellar-recruited behaviors. We treated female mouse pups subcutaneously with binge levels of EtOH during this developmental time window, and then aged these mice until P60, when a battery of behavioral assays, including locomotor, rotarod, and balance beam tests, was performed over a period of three weeks (Figure 1). We also harvested the cerebella of female littermates that had not undergone behavioral analysis on P105 in order to assay OL-related transcripts, which we have shown were dysregulated at P10 in a similar third trimester exposure mouse model [22].

**Figure 2:**
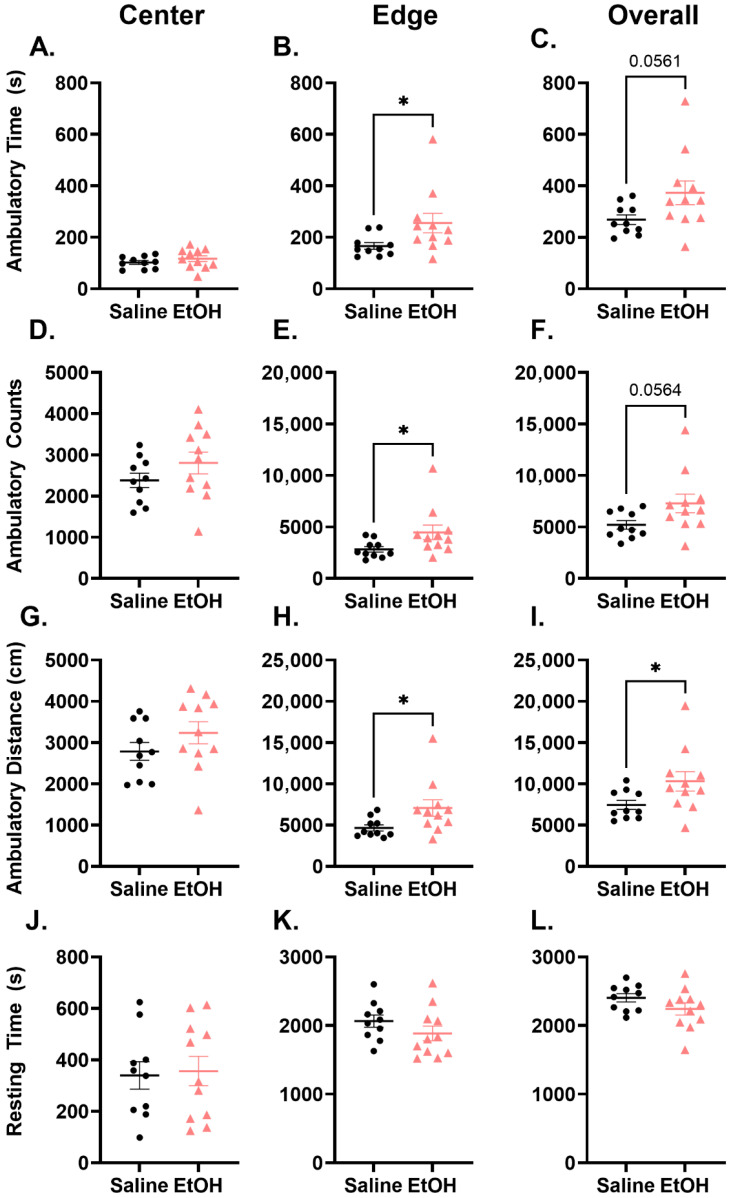
Open field test in adult females. Ambulatory time in the (**A**) center and (**C**) overall was not changed, but ambulatory time was increased in the (**B**) edge (t = 2.137, df = 19, *p* = 0.0459). Ambulatory counts were not changed in the (**D**) center or (**F**) overall, but there was an increase in the (**E**) edge (t = 2.108, df = 19, *p* = 0.0486). Ambulatory distance was not different in the (**G**) center but was increased (**I**) overall (t = 2.128, df = 19, *p* = 0.0466) and in the (**H**) edge (t = 0.0408, df = 19, *p* = 0.0408). There were no changes in resting time in the (**J**) center, (**K**) edge, or (**L**) overall. All points represent individual animals. N = 10–11. Unpaired *t*-test (* = *p* < 0.05).

To assess if third trimester equivalent EtOH exposure had long-term consequences on behavior, adult females underwent three behavioral assays: open field test (OFT) to assay locomotor changes, as well as rotarod and balance beam tests for cerebellar-recruited behaviors. In the open field test (OFT; Figure 2) we found that females exposed to EtOH during P4–9 had a significant increase in ambulatory distance overall in the chamber with a trend towards an increase in ambulatory time and counts (Figure 2C,F,I). These effects were driven by the significant increase in ambulatory time, counts, and distance in the edge of the chamber (Figure 2B,E,H), while there were no changes in locomotion in the center (Figure 2A,D,G). Despite changes in ambulatory behavior, there were no significant differences in the resting time in any location in the chamber or overall (Figure 2J–L), as well as in stereotypic time. Importantly, we also found that both saline- and EtOH-treated females habituated to the open field chamber over the 60 min period, suggesting that the overall changes in locomotor activity were not specific to a particular time period of testing (Supplementary Figure S1).

**Figure 3:**
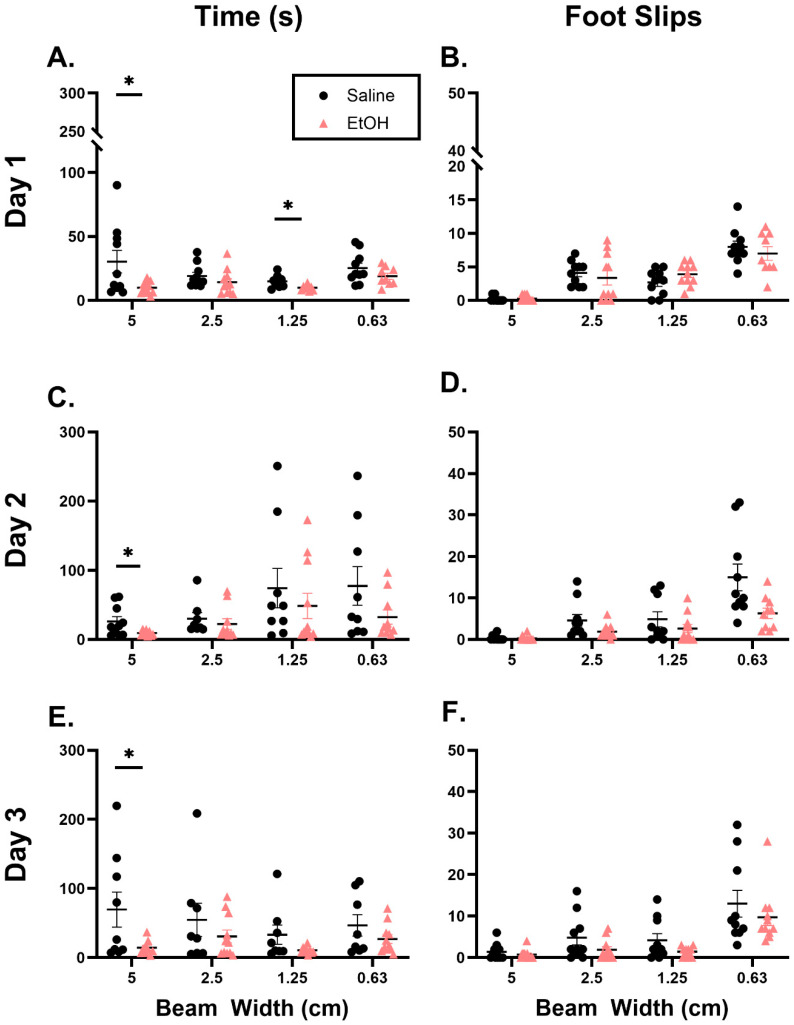
Balance beam. (**A**) On day 1, EtOH-exposed females spent significantly less time crossing the 5 cm beam (t = 2.392, df = 19, *p* = 0.0272) and the 1.25 cm beam (t = 2.574, df = 14, *p* = 0.0221). (**C**) On day 2, EtOH-exposed females took significantly less time to cross the 5 cm beam (t = 2.137, df = 16, *p* = 0.0484). (**E**) On day 3, EtOH-exposed females took significantly less time to cross the 5 cm beam (t = 2.153, df = 16, *p* = 0.0469). There were no changes in number of foot slips on (**B**) day 1, (**D**) day 2, or (**F**) day 3. N = 8–11. Individual points represent individual animals (* = *p* < 0.05). Unpaired *t*-tests were used for comparisons because of the need to exclude animals in individual assessments (see Section 2; Table 1).

**Figure 4:**
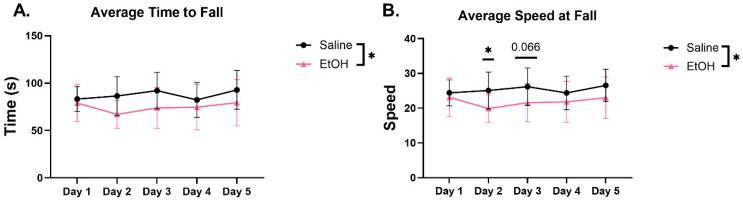
Rotarod. Ethanol-treated females had a significantly (**A**) lower time to fall (F(1,19)= 4.403, *p* = 0.0495) and (**B**) average speed (rpm) at fall (F(1,19) = 4.806, *p* = 0.0410) compared to saline-treated females across a five-day period. N = 10–11. Two-way ANOVAs with repeated measures and Tukey’s post hoc multiple comparison test have been used (* = *p* < 0.05).

After assessing locomotor activity, we probed for behaviors that are known to recruit the cerebellum, specifically balance beam [35] and rotarod [36]. On the balance beam test, we measured the time to cross the beam and the number of foot slips. EtOH-exposed females spent significantly less time to cross the 5 cm beam on days 1–3 and the 1.25 cm width beam on day 1 (Figure 3). However, these data showed a large level of variability and several statistical outliers were removed from the analysis (see Section 2; Table 1), making the results difficult to interpret. Despite the decrease in time to cross the 5 cm beam, there were no differences in the number of foot slips in any of the beam widths on any day (Figure 3). Over the 5 days of rotarod testing, females exposed to EtOH consistently fell earlier and at a lower speed than saline-exposed females, and there was a significant effect in treatment on the time to fall and speed at fall (Figure 4). Examining individual trials on each day showed similar effects, whereby both saline- and EtOH-exposed animals improved over the course of the three trials presented daily, but EtOH-exposed females fell earlier and at a lower speed across the majority of trials than saline mice (Supplementary Figure S2). Together, these behavior data suggest that EtOH exposure elicited subtle effects on locomotor activity and cerebellar-recruited behaviors, with a likely contribution from anxiety- and fear-mediated domains (see Section 4).

**Figure 5:**
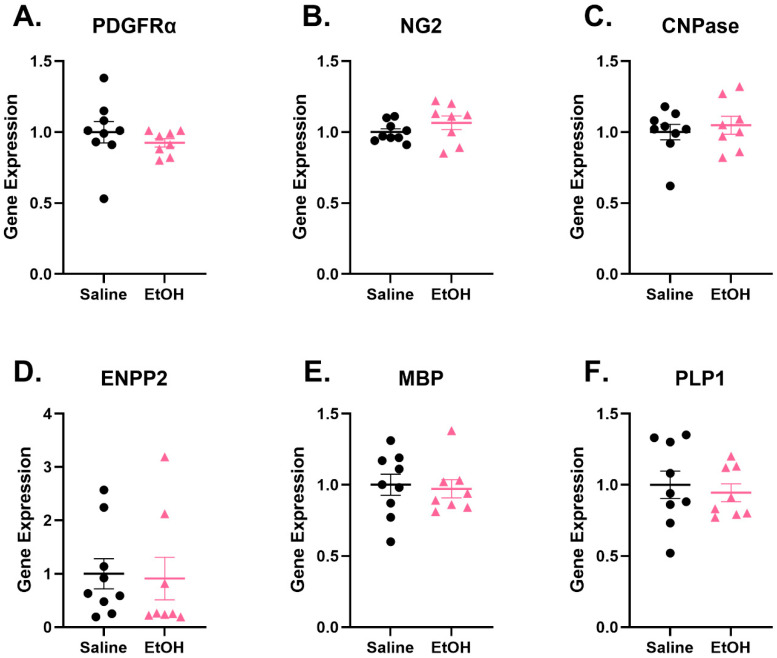
Oligodendrocyte lineage marker expression in the adult cerebellum. There were no changes in (**A**) PDGFRα, (**B**) NG2, (**C**) CNPase, (**D**) ENPP2, (**E**) MBP, or (**F**) PLP1 mRNA expression. Results are expressed as fold change in the ethanol group relative to the vehicle control group. Variance between groups was analyzed by Student’s *t*-test. n = 8–9.

Given that our female mice showed long-term behavioral consequences of early-life EtOH exposure, we sought to understand if this behavioral phenotype could be explained by long-term dysregulation in oligodendrocyte lineage (OL) transcripts. We had previously found that PDGFRα, NG2, CNPase, ENPP2, MBP, and PLP1 were downregulated 24 h following the final EtOH gavage treatment at P4–P9 in EtOH-exposed mice, while there were no changes in PDGFRα [22]. In order to determine how EtOH can have long-term effects on different OL transcripts, we quantified the expression of these six genes in the brains of behavior naïve mice on P105 (Figure 5). We found that, at P105, there were no differences in the expression of any of the probed transcripts between EtOH- and saline-exposed mice, suggesting that the effects observed at P10 in a similar model may not persist into adulthood. This suggested that long-term behavioral consequences of EtOH exposure may not be mediated by long-term changes in the transcriptomic profile of OL cells in this model.

## 4. Discussion

Our lab previously showed that EtOH exposure from P4 to P9 elicited subtle long-term effects on microglia dynamics and interactions with PCs in females but not males [30], resulting in a decrease in PC linear frequency only in females [24]. The goal of this project was to understand whether these cellular changes could manifest in behavioral alterations. To do this, we probed for cerebellar-recruited behaviors in adulthood in mice that had been exposed to EtOH on P4–9, the mouse equivalent of the human third trimester. We have reported that P4–9 in mice was a critical window for EtOH exposure in females when considering cerebellar-recruited behaviors. While we found long-term behavioral effects of early EtOH exposure, we found no changes in PDGFRα, NG2, CNPase, ENPP2, MBP, or PLP1 expression levels at P105 despite previously showing that these transcripts were downregulated 24 h following the final EtOH administration [22]. This suggested that persistent changes to the expression of these genes were unlikely to contribute to long-term behavioral deficits.

### 4.1. Behavioral Battery

The cerebellum is recruited for motor movements, learning, and coordination [37,38,39]. In this study, we investigated the effects of early-life EtOH treatment on locomotor, balance, and coordination behaviors using the open field test (OFT), balance beam, and rotarod test. In the OFT, we have reported an increase in ambulation observed only at the edge of the field, suggesting location-dependent hyperactivity. Location-dependent activity changes have been reported previously in CD1 mice that were perinatally exposed to EtOH [40], and prenatal ethanol exposure has been shown to result in an increase in cumulative movement, specifically in transgenic female mice [41]. In fact, we have previously reported edge-specific hyperactivity in adult male mice that were exposed to an environmental toxicant during neurodevelopment [42], further supporting a phenotype of long-term location-specific activity changes following developmental exposures. This location-dependent phenotype could be promoted by fear-mediated circuits or anxiolytic behaviors that can result in the mouse moving more in the dark, closed-in edge area. Further studies should include behavioral tests that specifically probe these domains.

Along with changes in locomotor activity, we have reported changes in motor behavior. Deficits in rotarod performance have been reported in mice exposed to EtOH prenatally, as well as in using prenatal and lactational exposure [43,44,45]. In our postnatal EtOH exposure model, we found a decreased latency to fall and speed at fall in EtOH-exposed mice in line with these previous findings. We also found a decrease in time to cross the balance beam compared to controls without a change in number of foot slips. This could reflect the OFT findings, whereby fear-mediated hyperactivity drove EtOH mice to run across the beam, while saline-exposed mice explored and habituated to the beam before crossing, especially because the differences were most evident on the 5 cm beam, which was the first test presented to the mice on each day. Considering both of these behavioral assays together, further studies are needed to probe anxiolytic- and fear-mediated domains, especially since children with FASDs can often exhibit increased anxiety [46]. In fact, some rodent behavioral studies have reported increases in anxiety-like behaviors using the elevated plus maze or O-maze [47,48,49], with some showing only male-specific deficits [50], which suggested sex-specific modulation of these behaviors.

Activity and motor domains, along with social and operant behaviors, have been previously investigated in FASD models. Motor skill learning, but not grip strength, has been reported to be impaired following prenatal EtOH exposure [41], and early postnatal EtOH exposure has been shown to result in female-specific deficits in adulthood in social tests and operant responses, while no effects were observed in males or females for anxiety-like behaviors [51]. Together, these data could suggest that multiple behavioral domains are vulnerable to EtOH exposure.

### 4.2. Oligodendrocyte Lineage Cells

Myelination has been described as a process perturbed in FASDs [7], and because we have observed EtOH-driven changes in oligodendrocyte lineage (OL) transcript expression in the cerebellum at P10 [22], we sought to understand if changes in OL transcript expression persisted into adulthood. In fact, we found that the decrease in expression of these transcripts did not persist. Although we found no changes in the expression of PDGFRα and NG2 (expressed by oligodendrocyte progenitor cells (OPCs)), CNPase and ENPP2 (expressed by pre-myelinating oligodendrocytes), or MBP and PLP1 (expressed by mature myelinating oligodendrocytes), it is possible that the quantity and quality of myelination in the cerebellum was affected by the early-life dysregulation of transcripts, as described in our previous study, which could have resulted in the improper development of the behavioral circuits probed in the behavioral experiments.

### 4.3. Sex-Specific Effects in FASDs

In the epidemiological literature, sex differences in neurobehavioral outcomes of FASDs have been reported [52,53], with an emerging understanding that males appeared to have more severe neurocognitive impairments, while females displayed more mood and anxiety disorders [53]. The evolution of symptoms also appeared to be sex-specific, with males showing earlier impairments in physical growth than females [54]. FASD mouse models have also revealed sex differences, with most showing more severe phenotypes in males across various physiological processes and organ systems [55,56]. While behaviorally EtOH-exposed males tended to have more impairments [52], one group has reported female-specific effects in social behaviors and operant conditioning [51], and we have previously reported female-specific effects in Purkinje cells [24]. These sex differences could be a result of the baseline sex differences in the cerebellum [57,58], potential differences in rates of ethanol metabolism [59], or interference of EtOH in the sexual differentiation of the brain by disrupting the endocrine system [60]. Because females are traditionally less studied in the context of neurodevelopmental insults and because our previous data showed female-specific cerebellar effects in our model [24], here we wanted to specifically address the effects of developmental EtOH exposure on behavior and OL cells; however, future studies should include both sexes to dissect sex-specific differences that arise as a result of developmental EtOH exposure.

### 4.4. Limitations and Future Directions

Evaluating EtOH’s effects on the third trimester has revealed potential mechanisms of FASD phenotypes, but it did not reveal the consequences of EtOH exposure throughout the entirety of neurodevelopment. In this study, we only evaluated exposures to EtOH in mice during the third trimester equivalent of cerebellar development in humans, and future studies should consider longer-term EtOH consumption that begins during the first and second trimesters, which could better mimic human developmental exposure. It is also important to note that even third trimester mouse models are heterogeneous, as they can use different exposure routes and different timings of administrations and doses. Our model, which used subcutaneous administration, may have different effects on the brain than models which relied on gavage, intraperitoneal, or vapor exposure. Similarly, we only investigated the effects of high-dose exposure, and future studies should also consider the effects of low-dose exposures that may be more common in the human population. We also only investigated OL transcripts in the cerebellum and cerebellar-recruited behaviors, leaving open the question of whether cerebellar myelination defects or defects in other cell types and brain regions underlie the behavioral deficits we observed and other behavioral deficits that likely exist. It is important to consider that the molecular assays occurred ~45 days after the behavioral assays. It is possible that expression of these transcripts at P105 does not accurately represent their expression at the time of the behavioral assays. Future experiments should assess molecular and behavioral endpoints in the same ages. Additionally, we focused on females, precluding an analysis of sex-specific phenotypes, which could shed light on male–female differences in EtOH’s effects on the brain.

## 5. Conclusions

This study demonstrated that early-life, binge-level EtOH exposure results in long-term locomotor, balance, and coordination consequences in females, despite the fact that EtOH-driven early changes in OL cell gene expression, which have been described in a similar model, are not apparent in adulthood.

## Figures and Tables

**Table 1:** Sample size in each experiment. Number of included animals and number of excluded animals (in parentheses).

Assay			Saline	EtOH
Open Field Test			10 (0)	11 (0)
Balance Beam	Day	Width (cm)		
	1	5	10 (0)	11 (0)
		2.5	10 (0)	11 (0)
		1.25	8 (2)	8 (3)
		0.63	10 (0)	9 (2)
	2	5	10 (0)	8 (3)
		2.5	8 (2)	10 (1)
		1.25	9 (1)	11 (0)
		0.63	10 (0)	10 (1)
	3	5	9 (1)	9 (2)
		2.5	9 (1)	11 (0)
		1.25	10 (0)	9 (2)
		0.63	8 (2)	11 (0)
Rotarod			10 (0)	11 (0)
qPCR			9 (0)	8 (0)

## Data Availability

The original contributions presented in this study are included in the article/Supplementary Materials. Further inquiries can be directed to the corresponding author.

## Supplementary Materials

The following supporting information can be downloaded at: https://www.mdpi.com/article/10.3390/cells15070608/s1, Figure S1: Open field test represented in five minute increments across the hour; Figure S2: Results from individual trials on each day of rotarod testing.
