# Supplementary material for: Early Postnatal Ethanol Exposure Has Long-Term Behavioral Consequences in Female Mice
Source: Cells. 2026 Mar 30;15(7):608. doi: 10.3390/cells15070608 (PMC13072417; doi:10.3390/cells15070608)
Supplement: Supplementary file 1 [file cells-15-00608-s001.zip › cells-4173767-supplementary.pdf]

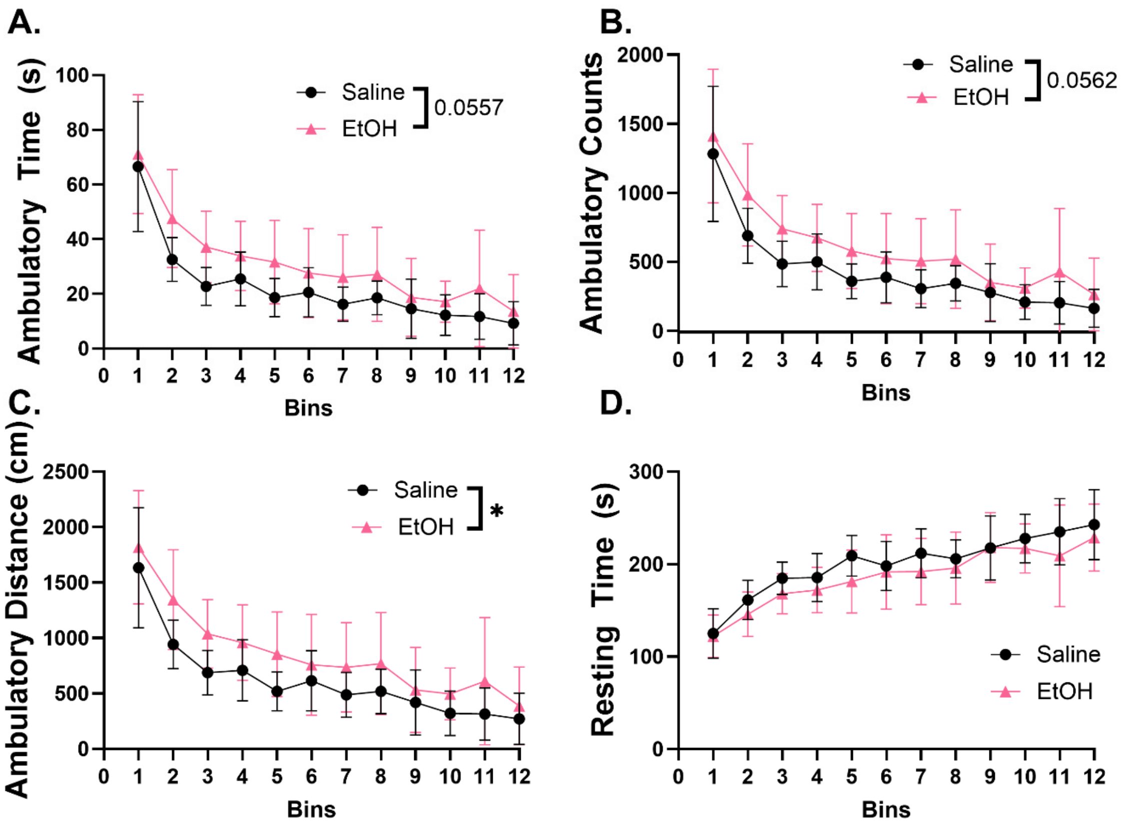

Supplementary Figure S1. Open field test represented in five minute increments across the hour. Saline and EtOH treated females showed habituation to the open field chamber with hyperactivity throughout the testing period in EtOH treated mice for (A) ambulatory time ($F(1,19)=4.154$, $p=0.0557$), (B) ambulatory counts ($F(1,19)=4.134$, $p=0.0562$), and (C) ambulatory distance ($F(1,19)=4.529$, $p=0.0466$). (D) Resting time showed habituation that was not significantly different between the two groups. N=10-11. Two-way ANOVA with repeat measures.

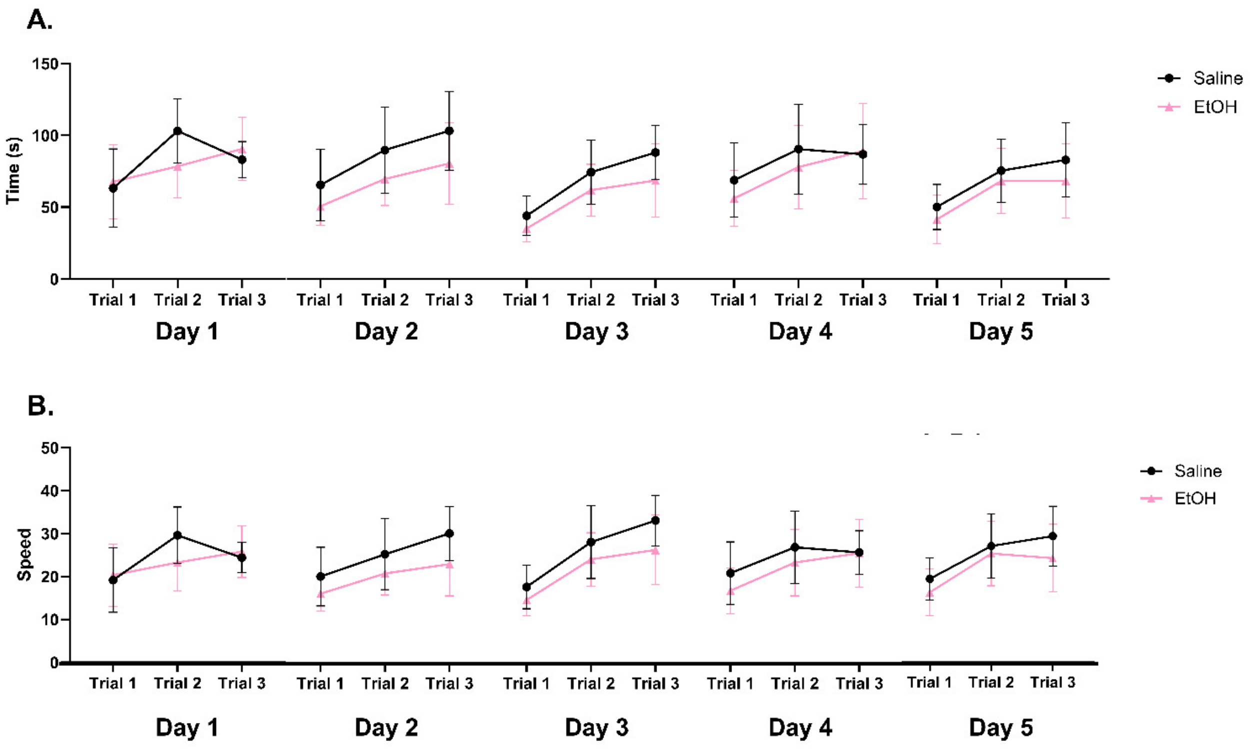

Supplementary Figure S2. Results from individual trial on each day of rotarod testing. Saline and EtOH treated females improved throughout the three trials presented each day both in the time to fall (A) and speed at fall (B).
